# Circulating Insulin-like Growth Factor Binding Protein-4 (IGFBP-4) is not Regulated by Parathyroid Hormone and Vitamin D in vivo: Evidence from Children with Rickets

**DOI:** 10.4274/jcrpe.v2i1.17

**Published:** 2010-12-08

**Authors:** Abdullah Bereket, Yaşar Cesur, Behzat Özkan, Erdal Adal, Serap Turan, Sertaç Hanedan Onan, Hakan Döneray, Teoman Akçay, Goncagül Haklar

**Affiliations:** 1 Division of Pediatric Endocrinology, Department of Pediatrics, Marmara University Medical Faculty, İstanbul, Turkey; 2 Division of Pediatric Endocrinology, Yüzüncü Yıl University Medical Faculty, Van, Turkey; 3 Atatürk University Medical Faculty, Erzurum, Turkey; 4 Ministry of Health Bakırköy Childhood and Maternity Education Hospital, İstanbul, Turkey; 5 Department of Biochemistry, Marmara University Medical Faculty, İstanbul, Turkey; +90 216 327 10 10/577+90 216 411 60 49abereket@e-kolay.netMarmara University Medical Faculty, Department of Pediatric Endocrinology - Tophanelioglu cad. Altunizade, İstanbul, Turkey

**Keywords:** Vitamin D, rickets, IGF-I, IGFBP-3, IGFBP-4, PTH, Bone

## Abstract

**Objective**: Insulin-like growth factor binding protein-4 (IGFBP-4), inhibits IGF actions under a variety of experimental conditions. Parathyroid hormone (PTH), 1.25-hydroxy(OH)vitamin D, IGF-I, IGF-II and transforming growth factor (TGF)-b are the major regulators of IGFBP-4 production in vitro. However, little is known about the in vivo regulation of circulating IGFBP-4 in humans.

**Methods**: We measured serum concentrations of calcium (Ca), phosphorus (P), alkaline phosphatase (ALP), PTH, vitamin D, IGF-I, IGFBP-3, and IGFBP-4 in infants (n=22) with nutritional rickets before and after treatment of rickets with vitamin D (300 000 U single dose po).

**Results**: The mean±SD age of the patients was 1.3±1.6 years (range 0.2-3). Serum Ca and P increased, whereas ALP and PTH decreased after treatment (Ca from 6.6±1.4 to 9.5±1.6 mg/dL, P from 3.9±1.4 to 5.4±0.8 mg/dL, ALP from 2590±2630 to 1072±776 IU/mL and PTH from 407±248 to 27.4±20.8 ng/dL, respectively). Vitamin D levels were low (7.8±2.5 ng/mL) and increased after treatment (18.1±4.0 ng/mL, p<0.001). Serum IGF-I and IGFBP-3 levels both increased after treatment (IGF-I: 13.5±12.2 vs. 23.7±14.2 ng/mL, p<0.001 and IGFBP-3: 1108±544 vs. 1652±424 ng/mL, p<0.001). However, serum IGFBP-4 levels did not change significantly after treatment (18.8±8.0 vs. 21.5±4.8 ng/mL). No correlation between PTH and IGF-I, IGFBP-3 or IGFBP-4 was detected. Significant correlations were observed between PTH and ALP (r=0.53, p<0.05), and between IGF-I and IGFBP-3 (r=0.46, p<0.05).

**Conclusion**: The results demonstrate that contrary to in vivo studies, circulating IGFBP-4 levels are not influenced by secondary hyperparathyroidism in vitamin D deficiency rickets since IGFBP-4 levels did not change after normalization of PTH with vitamin D treatment.

**Conflict of interest:**None declared.

## INTRODUCTION

In vitro and in vivo studies emphasize that insulin-like growth factor binding protein-4 (IGFBP-4) may play an important role in modulating IGF actions in bone. IGFBP-4 is the major IGFBP produced by human osteoblasts and has been shown to be a potent inhibitor of IGF-stimulated cell proliferation (1, 2). This effect is further modified by IGFBP-4 protease under variety of experimental conditions (3).Parathyroid hormone (PTH), 1, 25-hydroxy(OH)vitamin D, IGF-I, IGF-II and transforming growth factor (TGF)-β are known regulators of IGFBP-4 production in vitro in human bone cells (4, 5, 6, 7). However, little is known about the physiological regulation of circulating IGFBP-4 in humans. It was found that plasma IGFBP-4 levels correlated with bone mineral density in growth hormone (GH)-deficient adults (8). The increased circulating IGFBP-4 levels are found in elderly women with hip and spine fractures. Increase in IGFBP-4 was correlated with increased PTH in these women (9). This observation supported the role of PTH in the regulation of IGFBP-4. On the other hand, in subjects with primary hyperparathyroidism due to adenoma or hyperplasia, IGFBP-4 levels were found to be subnormal (10). Thus, human studies are inconclusive regarding the role of PTH in regulation of circulating IGFBP-4. To further investigate the role of PTH in regulation of IGFBP-4, we wanted to measure serum IGFBP-4 levels in situations, where hyperparathyroidism is more prominent and reversible. Vitamin D deficiency rickets is a perfect condition to test this hypothesis since secondary hyperparathyroidism is severe and is corrected rapidly after initiation of Vitamin D therapy. Thus, to investigate the role of PTH in the regulation of IGFBP-4 levels, we prospectively measured serum IGF-I, IGFBP-3, IGFBP-4 and PTH concentrations in infants with nutritional rickets before and after treatment.

## METHODS

Patients with rickets were recruited from the outpatient clinics of Pediatric Hospitals after obtaining informed consent from the parents/guardian of each child. Diagnosis of rickets was established according to clinical, biochemical and radiological findings. Children who had a history of prematurity, renal, liver or intestinal disease or evidence of any of these disorders on physical examination or laboratory testing were excluded. Altogether, 22 infants with a mean age of 1.3±1.6 years were included in the study. Blood samples were obtained before and 3 months after treatment of rickets with vitamin D. The treatment was achieved by giving a single oral dose of 300 000 U of Vitamin D (stoss-therapy). Serum from the blood samples was separated within 2 h of the collection and was stored at -20 °C until assayed. Serum calcium (Ca), phosphate (P), and total alkaline phosphatase (ALP) levels were determined by automatic analyzer. Serum intact PTH was measured by a two-site immunoradiometric assay (Allegro). Serum 25-(OH)Vitamin D levels were measured by chemiluminescence using Nichols Advantage competitive binding assay (San Juan Capistrano, California, USA). Serum IGF-I was determined by IRMA (DSL-5600 active, Diagnostics System laboratories, Webster, TX, USA) after separation of IGFs from IGFBPs by acid-ethanol extraction and neutralization as described previously (11). Including the extraction step, the intraassay coefficient of variation (CV) was 5%, whereas the interassay CV was 12 %. Serum concentrations of IGFBP-3 were also measured by IRMA (DSL-6600) (11). Intraassay CV was 6% and interassay CV was 16%. 

Serum IGFBP-4 was measured by an ELISA (DSL active IGFBP-4) assay according to the manufacturer’s directions. For all measurements, interassay variability was less than 9% and intraassay variability was less than 7%. All blood samples were measured in duplicate.

## STATISTICS

Paired t-test was used to evaluate the differences in parameters examined before and after treatment with Vitamin D. Simple regression was used to analyze the relationships between the study parameters.

## RESULTS

The mean (±SD) age of the patients was 1.3±1.6 (range 0.2 -3.0) years at the beginning of the study. Table 1 summarizes the findings on biochemical indices of rickets and IGF-I, IGFBP-3 and IGFBP-4 levels before and after treatment with vitamin D. These findings show that the patients had severe vitamin D deficiency with low blood levels of Ca and P, and very high levels of ALP and PTH. These values were almost completely normalized 3 months after treatment. Serum IGF-I and IGFBP-3 levels both increased after treatment for rickets, while serum IGFBP-4 levels did not change significantly (18.8±8.0 vs. 21.5±4.8 ng/ml). 

Correlation analyses demonstrated no relationship between PTH and IGF-I, IGFBP-3 or IGFBP-4 levels. Significant correlations were detected between PTH and ALP (r=0.53, p<0.05), and also between IGF-I and IGFBP-3 (r=0.46, p<0.05) before treatment, and before and after treatment combined.

**Table 1:**
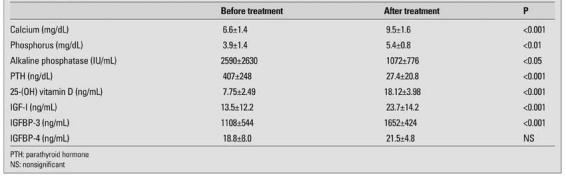
Serum calcium, phosphorus, alkaline phosphatase, PTH, 25-OH vitamin D, IGF-I, IGFBP-3 and IGFBP-4 levels before and after treatment in patients with rickets

## DISCUSSION

The results of this study demonstrate that circulating IGFBP-4 levels are not influenced by secondary hyperparathyroidism in vitamin D deficiency rickets since IGFBP-4 levels did not change after normalization of PTH with vitamin D treatment. This observation is contradictory to the data obtained in in vitro studies suggesting regulation of IGFBP-4 by PTH and/or Vitamin D.

In vitro studies demonstrated a stimulatory effect of PTH on IGFBP-4 production. Treatment of SaOS-2 cells with PTH for 3 hours caused a 3.3-fold increase in IGFBP-4 mRNA levels, which was determined by reverse transcription-polymerase chain reaction (7). 1.25 (OH)_2_ D3 increases the secretion of IGFBP-4 by human osteoblast-like cells (5). Data regarding regulation of serum IGFBP-4 in humans are scarce. Using Western ligand blot analysis, Rosen et al (9) showed that serum IGFBP-4 levels are higher in elderly women with hip fractures and elevated PTH levels compared with age-matched controls. It was speculated that an increased local production of IGFBP-4 would inhibit the IGF stimulatory actions on bone synthesis potentiating the effect of PTH on bone resorption in these patients. However, in a recent study, serum IGFBP-4 levels were positively correlated with only radial bone mineral density (BMD), but not with lumbar or femoral BMD and vertebral fractures (12). 

Honda et al (13) also found a weak correlation (r=0.26) between serum IGFBP-4 and PTH in healthy adults and in elderly individuals. They suggested that secondary hyperparathyroidism, which occurs as a consequence of age, could induce the inhibition of osteoblast proliferation by production of IGFBP-4 in the locale of bone-remodeling sites. Although much remains to be learned, our observation of no correlation in a more severe secondary hyperparathyroid state suggests that this conclusion is not valid. Consistent with our findings, Jehle et al (14), using Western blot found that IGFBP-4 levels in patients with primaryhyperparathyroidism are comparable to those in controls. Similarly, Van Doorn et al (10), using RIA, found subnormal IGFBP-4 levels in subjects with primary hyperparathyroidism due to adenoma or hyperplasia, supporting what we observed in a secondary hyperparathyroid state in the present study.

Unlike previous cross-sectional studies, the present study is the first one to prospectively analyze serum IGFBP-4 levels in a high PTH state and after PTH levels have been decreased. We have seen no significant change in IGFBP-4 levels after the dramatic reduction of PTH in the subjects, while a slight increase was observed in IGF-I and IGFBP-3 levels. It is likely that the observed correlation between IGFBP-4 and PTH in the previous cross-sectional studies was influenced by some covariants ,such as age. In fact, serum IGFBP-4 levels correlated more strongly with age than with PTH levels (13).

The increase in both IGF-I and IGFBP-3 levels after the treatment of rickets may be due to a direct stimulatory effect of vitamin D and/or to improvement of nutritional status. However, none of the patients in this series were in a malnourished state. Similar to our findings, increased consumption of milk in elderly subjects for 3 months resulted in a decline in both PTH and ALP by 9%, in a significant rise in IGF-I by 10% and in a nonsignificant fall in IGFBP-4 by 1.9% (15). This observation is consistent with the findings in our study, where IGFBP-4 levels did not change significantly despite dramatic decline in PTH and ALP levels. 

We conclude that circulating IGFBP-4 levels in children with rickets are not regulated by PTH or vitamin D, since the levels did not undergo significant change despite a 20-fold decrease in PTH levels after treatment with vitamin D. This finding is not in line with the data obtained from elderly persons and from in in vitro studies. It is likely that the paracrine and endocrine regulators of IGFBP-4 are different.
